# Relation between Established Glioma Risk Variants and DNA Methylation in the Tumor

**DOI:** 10.1371/journal.pone.0163067

**Published:** 2016-10-25

**Authors:** Anna M. Dahlin, Carl Wibom, Soma Ghasimi, Thomas Brännström, Ulrika Andersson, Beatrice Melin

**Affiliations:** 1 Department of Radiation Sciences, Oncology, Umeå University, Umeå, Sweden; 2 Department of Medical Biosciences, Pathology, Umeå University, Umeå, Sweden; National Cancer Institute, UNITED STATES

## Abstract

Genome-wide association studies and candidate gene studies have identified several genetic variants that increase glioma risk. The majority of these variants are non-coding and the mechanisms behind the increased risk in carriers are not known. In this study, we hypothesize that some of the established glioma risk variants induce aberrant DNA methylation in the developing tumor, either locally (gene-specific) or globally (genome-wide). In a pilot data set including 77 glioma patients, we used Illumina beadchip technology to analyze genetic variants in blood and DNA methylation in matched tumor samples. To validate our findings, we used data from the Cancer Genome Atlas, including 401 glioblastoma patients. Consensus clustering identified the glioma CpG island methylator phenotype (gCIMP) and two additional subgroups with distinct patterns of global DNA methylation. In the pilot dataset, gCIMP was associated with two genetic variants in *CDKN2B-AS1*, rs1412829 and rs4977756 (9p21.3, p = 8.1 x 10^−7^ and 4.8 x 10^−5^, respectively). The association was in the same direction in the TCGA dataset, although statistically significant only when combining individuals with AG and GG genotypes. We also investigated the relation between glioma risk variants and DNA methylation in the promoter region of genes located within 30 kb of each variant. One association in the pilot dataset, between the *TERT* risk variant rs2736100 and lower methylation of cg23827991 (in *TERT;* p = 0.001), was confirmed in the TCGA dataset (p = 0.001). In conclusion, we found an association between rs1412829 and rs4977756 (9p21.3, *CDKN2B-AS1*) and global DNA methylation pattern in glioma, for which a trend was seen also in the TCGA glioblastoma dataset. We also found an association between rs2736100 (in *TERT*) and levels of methylation at cg23827991 (localized in the same gene, 3.3 kbp downstream of the risk variant), which was validated in the TCGA dataset. Except for this one association, we did not find strong evidence for gene-specific DNA methylation mediated by glioma risk variants.

## Introduction

Glioma is a malignant brain tumor with few established risk factors. High doses of ionizing radiation increase the risk of developing glioma, whereas a personal history of allergy and diabetes reduce the risk [1,2]. Large genome-wide association studies (GWAS) and candidate gene studies have identified several common germline genetic variants (single nucleotide polymorphisms, SNPs) that are associated with risk of glioma [3–8]. Some of these variants are located in or nearby genes that are frequently altered by mutations and/or aberrant expression in the tumor, such as *EGFR*, *CDKN2A/B*, *TERT*, and *TP53*. Although the associations reported by GWAS are in many cases well established by replication in multiple studies, the mechanisms of action of these variants are generally poorly understood. The majority of SNPs that have been associated with risk of disease are located in introns or intergenic regions, and hence do not result in amino acid changes in transcribed proteins.

Aberrant DNA methylation is recognized as an important part of tumorigenesis in several malignancies. In glioma, subgroups of tumors with different patterns of DNA methylation have been described. One of these subgroups, the glioma CpG island methylator phenotype (gCIMP), is characterized by high levels of DNA methylation. gCIMP has been associated with specific molecular and clinical features, such as mutations in *IDH1* and *TP53*, lower grade, and a better outcome [9–11]. Turcan and collegues recently showed that a hypermethylated phenotype can be induced in primary human fibroblasts by expressing mutant *IDH1*, and suggested that this mutation is the molecular basis of gCIMP [12]. Compared to gCIMP, the other DNA methylation subgroups of glioma are less well defined.

The aim of this study was to investigate if established glioma risk variants are associated with global DNA methylation pattern of the tumor or with gene-specific promoter DNA methylation.

## Materials and Methods

### Study Subjects

Patients recruited to this study were diagnosed with a glioma at Umeå University Hospital, and have been previously described [13,14]. The majority of included subjects were diagnosed 2005–2008, whereas a few were diagnosed before this time period. All diagnoses were confirmed by pathology review. In total, 77 patients, from whom blood and tumor tissue were available, and for whom genotyping and methylation analyses were successful, were included in the final analysis. The majority of tumors were glioblastoma (77.9%), while lower grade astrocytoma and oligodendroglioma comprised 7.8% and 14.3%, respectively (Table 1). Ethical approval for the study was obtained from the Regional ethical review board in Umeå, section of medical science. Written consent to participate in the study was obtained from all subjects.

**Table 1:** Tumor characteristics.

	All^a^, n (% of column)	gCIMP, n (% of column)	Intermediately methylated, n (% of column)	Low methylation, n (% of column)	p-value^b^
Histological subtype					0.001453
Astrocytoma (grade II-III)	6 (7.8)	0 (0)	3 (8.8)	3 (8.8)	
Glioblastoma	60 (77.9)	3 (33.3)	28 (82.4)	29 (85.3)	
Oligodendroglioma	11 (14.3)	6 (66.7)	3 (8.8)	2 (5.9)	
Grade					1.526 x 10^−5^
II	7 (9.2)	6 (66.7)	1 (2.9)	0 (0)	
III	9 (11.8)	0 (0)	5 (14.7)	4 (12.1)	
IV	60 (78.9)	3 (33.3)	28 (82.4)	29 (87.9)	
Expression of mutated IDH1 (IHC)					1.591x 10^−7^
Negative	58 (86.6)	1 (12.5)	29 (93.5)	28 (100)	
Positive (>10% positive cells)	9 (13.4)	7 (87.5)	2 (6.5)	0 (0)	
Expression of p53 (IHC)					0.03089
Negative/faint/moderate	47 (71.2)	3 (37.5)	26 (83.9)	18 (66.7)	
Strong (≥70% positive cells)	19 (28.8)	5 (62.5)	5 (16.1)	9 (33.3)	
EGFR (FISH)					0.001434
Normal	7 (12.3)	1 (12.5)	1 (4.0)	5 (20.8)	
Chromosomal gain	31 (54.4)	7 (87.5)	9 (36.0)	15 (62.5)	
Amplification	19 (33.3)	0 (0)	15 (60.0)	4 (16.7)	
1p/19q co-deletion (FISH)					0.5019
Co-deleted	12 (22.2)	3 (37.5)	5 (21.7)	4 (17.4)	
Not co-deleted	42 (77.8)	5 (62.5)	18 (78.3)	19 (82.6)	
Ki-67 staining (IHC)					0.5461
<15% positive cells	29 (50.9)	3 (37.5)	12 (48.0)	14 (58.3)	
≥15% positive cells	28 (49.1)	5 (62.5)	13 (52.0)	10 (41.7)	
Homozygous deletion of *CDKN2A*					0.2286
1 or more copy	29 (44.6)	6 (75.0)	13 (39.4)	10 (41.7)	
Homozygous deletion	36 (55.4)	2 (25.0)	20 (60.6)	14 (58.3)	

^a^ Total n = 77. Missing values are present for grade (n = 1), IDH1 expression (n = 10), p53 expression (n = 11), EGFR amplification (n = 20), 1p/19q co-deletion (n = 23), Ki-67 (n = 20), and CDKN2A homozygous deletion (n = 12).

^b^ Fisher’s exact test

FISH, Fluorescence *in situ* hybridization; gCIMP, glioma CpG island methylator phenotype; IHC, immunohistochemistry.

### Selection of SNPs and genes

We selected 11 SNPs that have previously been associated with glioma risk in GWAS or candidate gene studies (including rs2736100, rs2252586, rs11979158, rs4295627, rs55705857, rs1412829, rs4977756, rs498872, rs78378222, rs6010620, and rs4809324; S1 Table) [3–8]. For analysis of gene-specific effects, we used UCSC genome/table browser to identify genes within 30 kbp from each SNP (http://genome.ucsc.edu/; Feb. 2009 (GRCh37/hg19) assembly). For SNPs with no genes within 30 kbp, the four closest genes were identified. In addition, we chose to include *MYC*, *CDKN2A*, and *CDKN2B* for their known involvement in tumorigenesis and location close to established glioma risk SNPs (although not within 30 kbp).

The promoter region of each gene was defined as 1500 bp upstream the transcription start site to 500 bp downstream the transcription start site. For genes with several transcripts, all transcripts with start sites more than 500 bp apart were included. All investigated genes are listed in S1 Table. For some genes, the methylation array had no CpG probes within the promoter. These genes were excluded from further analyses (S2 Table).

The chromosomal region 9p21.3 is homozygously deleted in a large proportion of glioblastoma [15]. Copy number variation (CNV) profiles of the tumors included in this study were established in a previous study [14] using the ASCAT algorithm, which gives information on CNV while accounting for the ploidy of the tumor and proportion of normal cells within the sample [16]. Based on CNV profiles, we identified tumors that were homozygously deleted in the promoter regions of *CDKN2A*, *CDKN2B*, *CDKN2B-AS1*, and *MTAP*, and excluded these tumors in analyses of gene-specific methylation in the respective genes (n = 25–35). In analyses of gene-specific methylation in the 9p21.3 region we also excluded 12 cases for which CNV profiles could not be determined.

### DNA extraction and Genotyping

Germline DNA was extracted from EDTA-venous blood samples using FlexiGene DNA Kit (Qiagen). Genotyping was performed by the SNP&SEQ Technology Platform, Uppsala, Sweden, using the HumanOmni1-Quad beadchip (Illumina). Five SNPs of interest (rs11979158, rs2252586, rs4295627, rs55705857, and rs78378222) were not represented on the chip and were therefore imputed using the software IMPUTE2 with data from the 1000 Genomes Project as the reference population [17]. The imputation info scores for the imputed SNPs were 0.997, 0.939, 0.999, 0.535, and 0.795, respectively, where values near 1 indicate that a SNP has been imputed with high certainty. Due to the low imputation info score (<0.85), rs55705857 (8q24.21) and rs78378222 (in *TP53*, 17p13.1) were excluded from further analysis.

### DNA methylation analyses

Tumor DNA was extracted from tumor tissue using QIAmp DNA Mini Kit (Qiagen) and bisulfite treated using the EZ-96 DNA Methylation Gold Kit (Zymo Research). Methylation was measured on Infinium HumanMethylation450 beadchips (Illumina), conducted by the SNP&SEQ Technology Platform in Uppsala, Sweden. The GenomeStudio software and the Methylation module (Illumina) were used to estimate the methylation level at each CpG site (the β-value, defined as (the methylated allele intensity) / (the methylated + unmethylated allele intensities+100) [18]). CpG probes with p detection value >0.05 were set as missing. CpG probes on the X and Y chromosomes, probes within 10 bp from a known SNP, and probes with >10% missing values were excluded from all analyses. To classify tumors by their global DNA methylation pattern, we used consensus clustering [19] based on the 8000 most variable CpG probes (standard deviation). Consensus clustering was originally described by Monti et al [19] and has been used by several studies investigating DNA methylation patterns in glioma [9,10,20]. In brief, this method repeats the clustering algorithm several times, sampling a proportion of individuals in each repetition. The output includes a “consensus matrix” which describes the proportion of clustering runs in which two individuals cluster together. We applied consensus clustering using the k-means algorithm (10 random starting sets, maximum of 1000 iterations) with 1000 repetitions, sampling 80% of individuals in each run, for k = 2–6. The choice of number of cluster for our data set was then made based on visual inspection of the consensus matrices for each k (S1A–S1E Fig). For each k, we also considered the change in the area under the empirical cumulative distribution curve, as described by Monti et al. [19] (S1F Fig). Consensus clustering was performed using the R package clusterCons (http://sourceforge.net/projects/clustercons/). Because the clustering analyses allowed no missing values, missing values were replaced with the median value across all individuals in these analyses.

### Immunohistochemistry (IHC) and Fluorescence *in situ* hybridization (FISH)

Immunohistochemical staining of tumor tissue using primary monoclonal anti-P53 (DO-7), anti-IDH1 (R132H), and anti-Ki-67 (30–9) antibodies and evaluation of 1p/19q co-deletion and EGFR amplification using FISH has previously been described in detail [13].

### TCGA data

To validate our findings, we used data from 401 glioblastoma patients in the TCGA database (http://cancergenome.nih.gov/) [10,15,20]. Before consensus clustering and analyses of gene-specific promoter methylation, TCGA subjects were divided into two non-overlapping groups; 116 subjects with methylation data from the Infinium HumanMethylation450 beadchip, and 285 subjects with methylation data from the Infinium HumanMethylation27 beadchip. Consensus clustering of TCGA subjects was based on the same CpG probes as for consensus clustering of the 77 patients in our pilot dataset. Notably, only 292 CpG probes were overlapping between the HumanMethylation27 chip and the 8000 most variable CpG probes in the pilot dataset. To test the concordance between the two separate clustering analyses of TCGA subjects, we selected six TCGA samples analyzed on the 450k methylation array (random selection of two samples from each DNA methylation cluster). These six samples were then clustered together with the 285 samples analyzed on the 27k array. In these analyses, all six samples were assigned the same DNA methylation cluster (low, intermediately, or highly methylated) as when clustered together with the other 110 samples analyzed on the 450k array.

TCGA subjects were genotyped on the Illumina 550K Infinium HumanHap550 SNP array or the Affymetrix Genome-Wide Human SNP Array 6.0. Five SNPs of interest (rs4295627, rs1412829, rs4977756, rs6010620, and rs4809324) were not genotyped by the Affymetrix array. These SNPs were imputed as described above (imputation info scores = 0.960, 0.990, 0.959, 0.898, and 0.825, respectively). Imputed rs4809324 genotypes were excluded from further analyses due to low imputation info score (<0.85).

The relation between DNA methylation and *TERT* mRNA expression was investigated in a subset of TCGA subjects analysed on the 450k methylation array for whom mRNA expression data from the Affymetrix HG-U133 array was available.

### Statistical analyses

Association between genome-wide methylation pattern in the tumor and tumor characteristics (histological subtype, grade, protein expression, DNA amplification/deletion) was assessed using Fisher’s exact test. Association between germline genetic variants and genome-wide methylation pattern in the tumor was assessed using Chi-Square test or Fisher’s exact test (when expected sample count in a table cell was <5). Association between germline genetic variants and local DNA methylation in the tumor was investigated using Kruskal-Wallis rank sum test. The relation between DNA methylation and gene expression was estimated using Spearman’s rho. Statistical calculations and data management were done using R (version 3.0.0, http://www.R-project.org) [21] and PLINK (version 1.07, http://pngu.mgh.harvard.edu/purcell/plink/) [22]. All genomic positions are based on the GRCh37/hg19 assembly.

## Results

The association between tumor DNA methylation and germline glioma risk variants was investigated in a pilot dataset including 77 glioma patients. Diagnoses included astrocytoma grade II-III (n = 6), glioblastoma (n = 60), and oligodendroglioma (n = 11). Patient and tumor characteristics are described in Table 1. Associations with p<0.05 in the pilot dataset were shortlisted for validation in 401 TCGA glioblastoma patients.

### Global DNA methylation

**Fig 1:**
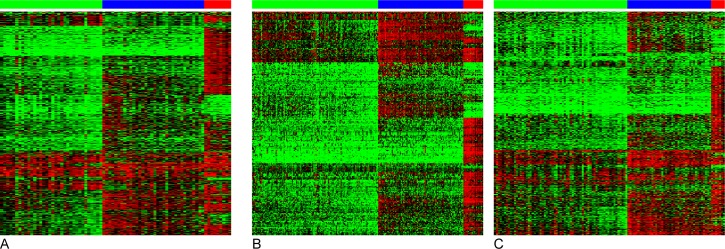
Consensus clustering reveals three distinct tumor subgroups. Consensus clustering of the 8000 most variable methylation-probes in the pilot dataset identified three subgroups of tumors with low (green bar), intermediate (blue bar), and high levels of DNA methylation (gCIMP, red bar). The average β-value for each tumor and CpG probe range from 0 (absence of methylation, green in heatmap) to 1 (complete methylation, red in heatmap). Consensus clustering was performed separately in (A) 77 glioma tumors analyzed on the 450k methylation array, (B) 285 TCGA glioblastoma tumors analyzed on the 27k methylation array and (C) 116 TCGA glioblastoma tumors analyzed on the 450 k methylation array.

Based on results from consensus clustering (S1 Fig), we chose to classify tumors into three subgroups based on their global DNA methylation patterns (Fig 1). 34 tumors (44.2%) displayed low levels of DNA methylation, 34 tumors (44.2%) displayed intermediate levels, and 9 tumors (11.7%) displayed markedly higher levels. The markedly higher DNA methylation levels are indicative of gCIMP [10]. In the pilot dataset, gCIMP status was associated with *IDH1* mutation (p = 1.6 x 10^−7^), oligodendroglial histopathology (p = 0.0015), and strong expression of p53 (indicative of mutation[23], p = 0.031), and was inversely associated with tumor grade (p = 1.53 x 10^−5^) and EGFR amplification (p = 0.0014) (Table 1).

**Fig 2:**
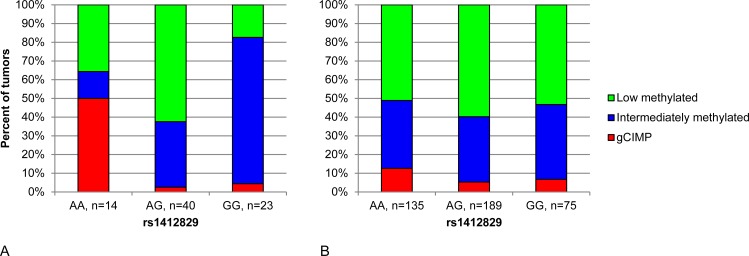
Glioma risk variant rs1412829 (*CDKN2B-AS1*) is associated with DNA methylation pattern of the tumor. Distribution of DNA methylation patterns between genotypes in (A) 77 glioma tumors (the pilot dataset), and (B) 399 TCGA glioblastoma tumors. P_AA vs. AG/GG_ = 2.59 x 10^−5^ and 0.048 in (A) and (B), respectively.

We then investigated the relation between global DNA methylation pattern in the tumor and 9 established glioma risk variants (S3 Table). Two variants, rs1412829 and rs4977756, both located on chromosome 9p21.3, were associated with the global DNA methylation pattern of the tumor (p = 8.07 x 10^−7^ and 4.81 x 10^−5^, respectively). An overrepresentation of gCIMP tumors was seen in patients with the rs1412829 AA (non-risk) genotype. 50% of individuals with the AA genotype had a gCIMP tumor, compared to 2.5% and 4.3% of patients with rs1412829 AG and GG genotypes, respectively (Fig 2). rs1412829 and rs4977756 are in linkage disequilibrium (D’ = 0.89; r^2^ = 0.74 [13]), and the relation between rs4977756 and global DNA methylation pattern was similar to that described for rs1412829 (S2 Fig). An overrepresentation of gCIMP tumors among individuals with the rs1412829 AA genotype was seen also when restricting analyses to glioblastoma patients (n = 60; p = 4.18 x 10^−4^; S3 Fig) or non-glioblastoma patients (n = 17; p = 0.002; S3 Fig). None of the other seven investigated glioma risk variants were associated with the global DNA methylation pattern of the tumor (all p>0.05; S3 Table).

Of the 8000 most variable CpG probes in the pilot dataset, 6873 and 292 passed quality control (<10% missing values) in the TCGA datasets analyzed on the 450k and 27k methylation arrays, respectively. These CpG probes were used for consensus clustering to identify TCGA tumors with high (gCIMP), intermediate, and low levels of global DNA methylation (Fig 1). The distribution of tumors with high, intermediate and low DNA methylation levels was not statistically significantly different between individuals with rs1412829 AA, AG and GG genotypes in the TCGA dataset (p = 0.137; S3 Table; Fig 2). An overrepresentation of gCIMP tumors among individuals with the rs1412829 AA genotype was observed in the TCGA dataset when combining individuals with AG and GG genotypes (p_AA vs. AG+GG_ = 0.048). When combining the pilot and TCGA data sets, we observed a p-value of 4.35 x 10^−4^ for the difference in the distribution of tumors with high, intermediate and low DNA methylation between individuals with AA vs AG/GG genotypes.

### Gene-specific (local) promoter methylation

**Fig 3:**
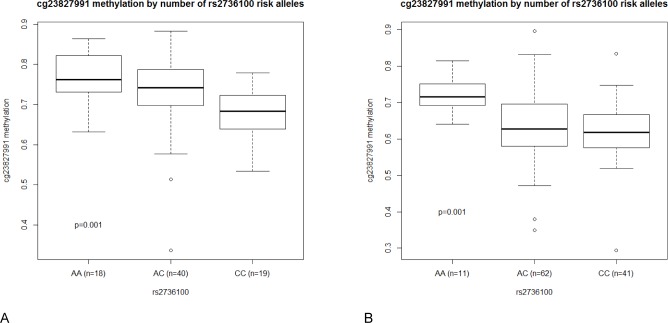
Levels of cg23827991 methylation by rs2736100 genotype. (A) 77 glioma tumors (the pilot dataset) and (B) 115 TCGA glioblastoma tumors analyzed on the 450 k methylation array.

For each glioma risk SNP, we identified 1–6 genes in the same genetic region (S1 Table). We then compared the levels of promoter methylation in individuals carrying no, one, or two copies of the established risk allele. (Table 2 and S4 Table). In total, we tested 199 combinations of SNPs and methylation probes. For 13 combinations, the SNP was associated with methylation of the nearby CpG probe (p<0.05; p-values not corrected for multiple testing; Table 2). Eleven of these associations were investigated in the TCGA dataset, whereas two combinations of SNPs and CpG probes could not be tested because imputation of the SNP failed or the CpG probe failed or was not represented on the methylation arrays. The rs2736100 genotype was associated with methylation at cg23827991 in the pilot data set (p = 0.001) as well as the TCGA 450k dataset (p = 0.001) (Fig 3; Table 2; p-values not corrected for multiple testing). In general, carriers of the rs2736100 risk allele (C) had lower methylation at cg23827991. This CpG probe and this SNP are both localized in *TERT*. None of the other nine investigated SNP/CpG probe combinations were statistically significant in the TCGA datasets.

**Table 2:** Associations between glioma risk SNPs and CpG probe methylation with p<0.05 in the pilot dataset.

SNP (gene)	risk allele	CpG probe (promoter region^a^)	dataset	β-value^b^, mean			p^c^
				no risk allele	1 risk allele	2 risk alleles	
rs2736100 (*TERT*)	C	cg23827991 (*TERT*)	pilot	0.77	0.73	0.68	0.001
			TCGA (450k)	0.72	0.63	0.62	0.001
rs4295627 (8q24.21^d^)	G	cg03003858 (*MYC*)	pilot	0.01	0.00	0.00	0.007
			TCGA (450k)	0.02	0.01	0.01	0.272
rs4295627 (8q24.21^d^)	G	cg24666276 (*MYC*)	pilot	0.10	0.13	0.10	0.019
			TCGA (450k)	0.13	0.12	0.09	0.147
rs4809324 (*RTEL1*)	G	cg05358404 (*RTEL1*)	pilot	0.68	0.63	0.87	0.042
			TCGA (27k)	0.65	0.67	0.49	0.296
rs4809324 (*RTEL1*)	G	cg16246590 (*ARFRP1*, *ZGPAT*)	pilot	0.01	0.02	0.02	0.038
			TCGA^e^	n.a.	n.a.	n.a.	n.a.
rs498872 (*PHLDB1*)	A	cg04541078 (*ARCN1*)	pilot	0.06	0.05	0.05	0.019
			TCGA (27k)	0.04	0.04	0.03	0.277
rs6010620 (*RTEL1*)	G	cg03873930 (*ARFRP1*)	pilot	0.50	0.76	0.74	0.022
			TCGA (450k)	0.80	0.72	0.74	0.699
			TCGA (27k)	0.65	0.64	0.66	0.688
rs6010620 (*RTEL1*)	G	cg04078896 (*ZGPAT*)	pilot	0.11	0.24	0.21	0.021
			TCGA (450k)	0.23	0.19	0.19	0.537
			TCGA (27k)	0.14	0.16	0.16	0.968
rs6010620 (*RTEL1*)	G	cg07382590 (*STMN3*)	pilot	0.00	0.01	0.01	0.005
			TCGA (450k)	0.02	0.02	0.02	0.98
rs6010620 (*RTEL1*)	G	cg14862171 (*STMN3*)	pilot	0.88	0.91	0.90	0.016
			TCGA^e^	n.a.	n.a.	n.a.	n.a.
rs6010620 (*RTEL1*)	G	cg18611245 (*ZGPAT*)	pilot	0.16	0.27	0.24	0.038
			TCGA (450k)	0.26	0.26	0.23	0.472
			TCGA (27k)	0.20	0.23	0.23	0.879
rs6010620 (*RTEL1*)	G	cg20642413 (*ZGPAT*)	pilot	0.18	0.31	0.30	0.043
			TCGA (450k)	0.35	0.28	0.28	0.36
rs6010620 (*RTEL1*)	G	cg21953717 (*RTEL1-TNFRSF6B*)	pilot	0.85	0.94	0.95	0.032
			TCGA (450k)	0.95	0.93	0.92	0.188

^a^ The promoter region was defined as the region 1500 bp upstream the transcription start site to 500 bp downstream the transcription start site.

^b^ The β-value range from 0 to 1 for each cpg probe and tumor sample, where 0 indicates the absence DNA methylation and 1 indicates complete DNA methylation.

^c^ Kruskal-Wallis rank sum test

^d^ No gene within 30 kbp

^e^ This combination of methylation-probe and SNP could not be tested in the TCGA dataset due to failure of imputation of the SNP and/or the methylation probe failed or was not represented on the methylation array.

The relation between methylation at cg23827991 and *TERT* mRNA expression was investigated in 67 TCGA subjects for whom these two data types were available. We found an indication of a negative association, however not statistically significant, between cg23827991 and *TERT* mRNA expression (p = 0.07, Spearman’s rho = -0.22).

## Discussion

A number of glioma susceptibility loci have been identified by GWAS and candidate gene studies [3–8], but relatively little is known about the mechanisms through which these loci increase the risk of glioma. In this study we investigated the hypothesis that some of the established glioma risk SNPs have an effect on DNA methylation in the tumor, either locally through promoter methylation of nearby genes, or globally.

We classified tumors into one of three groups based on patterns of global DNA methylation; gCIMP (highly methylated), intermediately methylated, or low methylated tumors. A problem when using clustering algorithms to classify tumors into subgroups is that often the “true” number of subgroups present in a dataset is not known. Our decision to classify tumors into three subgroups was based on our findings from consensus clustering, which indicated that clustering tumors into more than three subgroups introduced spurious clusters that varied with random sampling. The finding of three distinct patterns of global DNA methylation is in line with the original TCGA report on DNA methylation in glioblastoma [10]. The numbers of methylation subgroups reported in other studies have varied along with variables such as inclusion of pediatric cases, the proportion of lower grade glioma, the methods used to measure methylation, and the clustering algorithms employed [9–11,20,24–28]. In line with findings from previous studies, gCIMP status of the tumor was associated with IDH1 mutation, lower tumor grade, histopathology of the tumor, lack of EGFR amplification, and strong p53 staining (indicative of mutation [23]) [9–11,27].

Of the nine SNPs we investigated, two were associated with the global DNA methylation pattern of the tumor, rs4977756 and rs1412829, located on chromosome 9p21.3. These two SNPs are in linkage disequilibrium and likely represent an association between DNA methylation and one variant in the region, rather than two independent associations. For both SNPs, we observed an overrepresentation of gCIMP tumors among patients carrying the non-risk homozygous (AA) genotype (Fig 2). We made the same observation in the TCGA dataset, although differences were smaller and statistically significant only when combining individuals with AG and GG genotypes. TCGA tumors were exclusively glioblastoma, whereas the pilot dataset comprised glioblastoma as well as lower grade astrocytoma and oligodendroglioma tumors. However, because our finding was observed in the pilot data set also when restricting analyses to glioblastoma, the inclusion of low-grade tumors does not account for the whole difference between results in the two data sets. Since our pilot data set is small, and differences were smaller in the TCGA data set, it is possible that the observation of overrepresentation of gCIMP tumors among patients carrying the rs1412829 and/or rs4977756 non-risk homozygous (AA) genotypes is a chance finding.

The genetic variants rs1412829 and rs4977756 are both located on chromosome 9p21.3, within the long non-coding RNA *CDKN2B-AS1* (a.k.a. *ANRIL*). *CDKN2B-AS1* has been suggested to interact with polycomb repressive complex-1 and 2 in the regulation of epigenetic transcriptional repression of *CDKN2A* and *CDKN2B* through mechanisms involving histone modifications [29]. *CDKN2A* and *CDKN2B* are localized in the same region as *CDKN2B-AS1*, and are coding for tumor suppressor proteins p16INK4a/p14ARF and p15, respectively. This genetic region also contains susceptibility loci for a number of different malignancies and other diseases [30] and is a region that is frequently affected by somatic alterations in tumors, including glioma [15]. We found no evidence for an association between the two glioma risk SNPs in the 9p21.3 region and DNA methylation of *CDKN2A* or *CDKN2B*. However, homozygous deletion of the chromosomal region 9p21.3, harbouring *CDKN2A*, *CDKN2B*, *CDKN2B-AS1*, and *MTAP*, is a frequent event in glioblastoma [15]. When investigating gene-specific methylation in these genes, we excluded data from 25–35 tumors with homozygous deletion of these genes. Therefore, the investigations of these genes suffer from particularly low power.

When investigating the relation between glioma risk variants and DNA methylation in promoter regions of nearby genes, we found an association between a glioma risk variant localized in *TERT*, rs2736100, and lower methylation of cg23827991, a CpG probe localized close to the first exon of an alternative *TERT* transcript (uc003jbz.1). The association between rs2736100 and methylation at cg23827991 has previously been described in other tissue types, including breast, kidney, and both normal and tumor tissue from lung [31–33]. In a previous study of glioblastoma samples, none of the studied SNPs in the *TERT* (5p15.33) region were associated with nearby DNA methylation [34]. Glioma risk SNP rs2736100, or SNPs in strong linkage disequlibrium with rs2736100, were however not investigated.

The rs2736100 C allele has been associated with a higher mRNA expression of TERT when measured in gastric cancer, lung cancer and esophageal squamous cell carcinoma as well as normal tissue adjacent to lung and esophageal squamous cell carcinoma [35–37]. RNA expression data to investigate the relation between *TERT* promoter methylation and *TERT* expression was not available for our pilot set of gliomas. In a limited set of TCGA subjects, we found a trend of a higher *TERT* mRNA expression in subjects with lower levels of cg23827991 methylation. In a previous study, Nagarajan et al. found a genomic region, just upstream of the first exon on an alternative *TERT* transcript, that was hypomethylated in three of five glioblastoma tumors. They also described elevated expression of an alternative *TERT* transcript in glioblastoma samples compared to normal brain [38].

A limitation of the present study was the relatively large number of tests performed to assess associations between genetic risk variants and nearby gene promotor methylation in our pilot data (199 SNP/CpG probe combinations), resulting in a large risk of type I errors (false positives). However, at the initial stage of the analyses, our main concern was to avoid type II errors (false negatives). Considering that significant (p<0.05) associations from the pilot phase were further validated in the independent TCGA dataset, the overall risk of our findings being false positives was reduced. Due to failure of genotype imputation or lack of coverage on the methylation arrays, we could not validate two of 13 associations between genetic risk variants and CpG probe-specific methylation that was found in the pilot dataset. To reduce the number of statistical tests, we limited our investigations to CpG probes within the gene promoter region, which was defined as 1500 base pair upstream to 500 bp downstream the transcription start site. This definition has previously been used by Sturm et al. to find gene promoters that are differently methylated between glioma subgroups [9].

The glioma risk SNPs selected for investigation in this study are well established through investigations in large and independent populations. Although genotyping arrays over time have included increasing numbers of variants, it is important to note that the strongest associations reported by GWAS are signals of an association in the region, and do not necessarily come from the true functional variants. The presence of yet undiscovered, functional variants that are associated with local or global DNA methylation is still possible.

In conclusion, we observed an overrepresentation of gCIMP tumors in patients with the rs1412829 AA (non-risk) genotype (located in *CDKN2B-AS1*), which was present both in the pilot dataset and the TCGA dataset, although statistically significant in the latter only when combining individuals with AG and GG genotypes. We also found an association between glioma risk variant rs2736100 (*TERT*) and reduced methylation of CpG probe cg23827991 (*TERT*). Apart from this, we did not find strong support for our hypothesis that glioma risk variants affect DNA promoter methylation of adjacent genes. To confirm these findings, a larger study, including glioma patients with tumors of different histological subtypes and grade, is needed.

## Supporting Information

S1 Fig Consensus clustering. Consensus clustering performed on the 8000 most variable CpG probes using the k-means algorithm in 1000 repetitions on 80% of individuals. (A-E) Consensus matrices for k = 2–6, illustrating the number of times two individuals cluster together on a scale from blue (rarely) to red (the majority of times). (F) Delta-k plot indicating that, based on DNA methylation in the 8000 most variable CpG probes, tumors can be divided into three distinct subgroups.
(PDF)

S2 Fig Association between rs4977756 and DNA methylation pattern of the tumor. Distribution of DNA methylation patterns between genotypes in (A) 77 glioma tumors, and (B) 392 TCGA glioblastoma tumors. P_AA vs. AG/GG_ = 4.41 x 10^−4^ and 0.23 in (A) and (B), respectively.
(PDF)

S3 Fig Association between rs1412829 and DNA methylation pattern in glioblastoma and non-glioblastoma. (A) Glioblastoma (n = 60) and (B) non-glioblastoma (lower grade astrocytoma and oligodendroglioma; n = 17) tumors in the pilot dataset.
(PDF)

S1 Table All investigated SNPs and genes. (DOCX)

S2 Table Genes with no CpG probes in the promoter region. (DOCX)

S3 Table Associations between glioma risk SNPs and global DNA methylation. (DOCX)

S4 Table Associations between glioma risk SNPs and CpG site methylation in nearby gene promoters. (DOCX)
