# Supplementary material for: Relation between Established Glioma Risk Variants and DNA Methylation in the Tumor
Source: PLoS One. 2016 Oct 25;11(10):e0163067. doi: 10.1371/journal.pone.0163067 (PMC5079592; doi:10.1371/journal.pone.0163067)
Supplement: S1 Fig — Consensus clustering performed on the 8000 most variable CpG probes using the k-means algorithm in 1000 repetitions on 80% of individuals. (A-E) Consensus matrices for k = 2–6, illustrating the number of times two individuals cluster together on a scale from blue (rarely) to red (the majority of times). (F) Delta-k plot indicating that, based on DNA methylation in the 8000 most variable CpG probes, tumors can be divided into three distinct subgroups. (PDF) [file pone.0163067.s001.pdf]

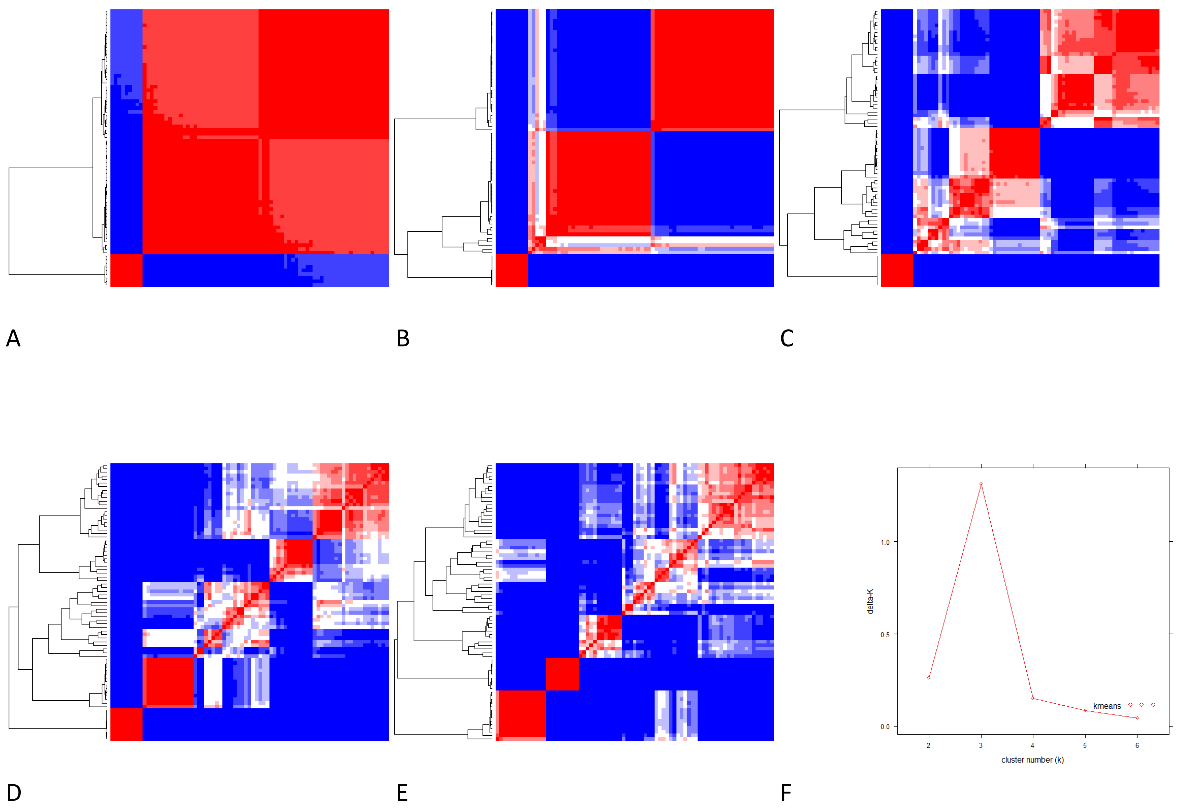

S1 Figure. Consensus clustering. Consensus clustering performed on the 8000 most variable CpG probes using the k-means algorithm in 1000 repetitions on 80% of individuals. (A-E) Consensus matrices for k=2-6, illustrating the number of times two individuals cluster together on a scale from blue (rarely) to red (the majority of times). (F) Delta-k plot indicating that, based on DNA methylation in the 8000 most variable CpG probes, tumors can be divided into three distinct subgroups.
