# Supplementary material for: Relation between Established Glioma Risk Variants and DNA Methylation in the Tumor
Source: PLoS One. 2016 Oct 25;11(10):e0163067. doi: 10.1371/journal.pone.0163067 (PMC5079592; doi:10.1371/journal.pone.0163067)
Supplement: S2 Fig — Distribution of DNA methylation patterns between genotypes in (A) 77 glioma tumors, and (B) 392 TCGA glioblastoma tumors. PAA vs. AG/GG = 4.41 x 10^−4^ and 0.23 in (A) and (B), respectively. (PDF) [file pone.0163067.s002.pdf]

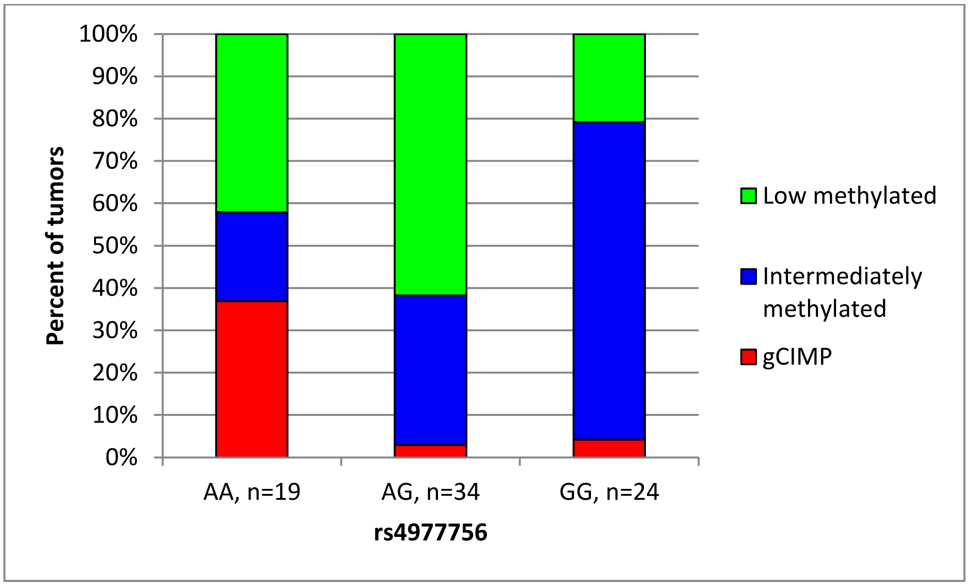

A

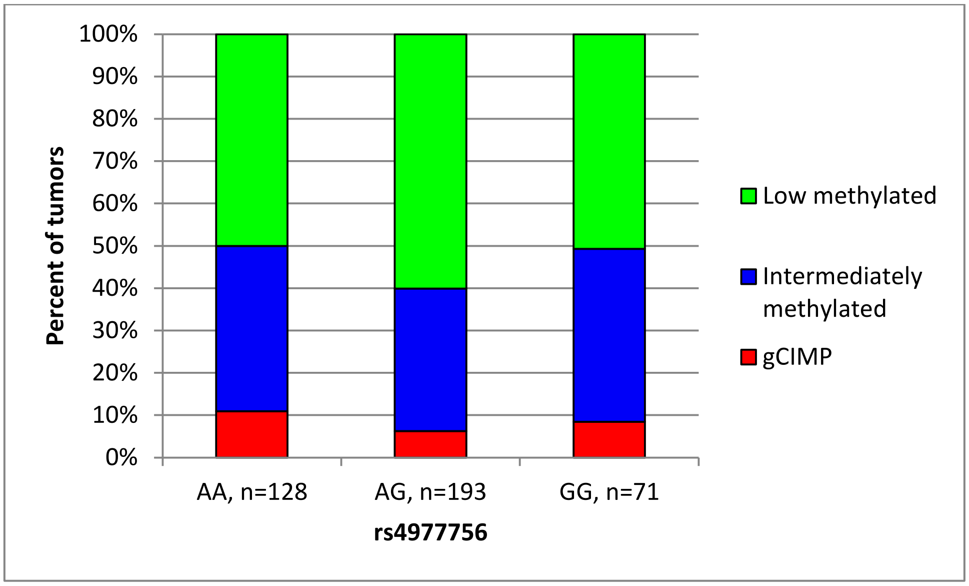

B

S2 Figure. Association between rs4977756 and DNA methylation pattern of the tumor. Distribution of DNA methylation patterns between genotypes in (A) 77 glioma tumors, and (B) 392 TCGA glioblastoma tumors. $P_{AA\ vs.\ AG/GG}$=4.41 x 10^{-4} and 0.23 in (A) and (B), respectively.
