# Supplementary material for: Relation between Established Glioma Risk Variants and DNA Methylation in the Tumor
Source: PLoS One. 2016 Oct 25;11(10):e0163067. doi: 10.1371/journal.pone.0163067 (PMC5079592; doi:10.1371/journal.pone.0163067)
Supplement: S3 Fig — (A) Glioblastoma (n = 60) and (B) non-glioblastoma (lower grade astrocytoma and oligodendroglioma; n = 17) tumors in the pilot dataset. (PDF) [file pone.0163067.s003.pdf]

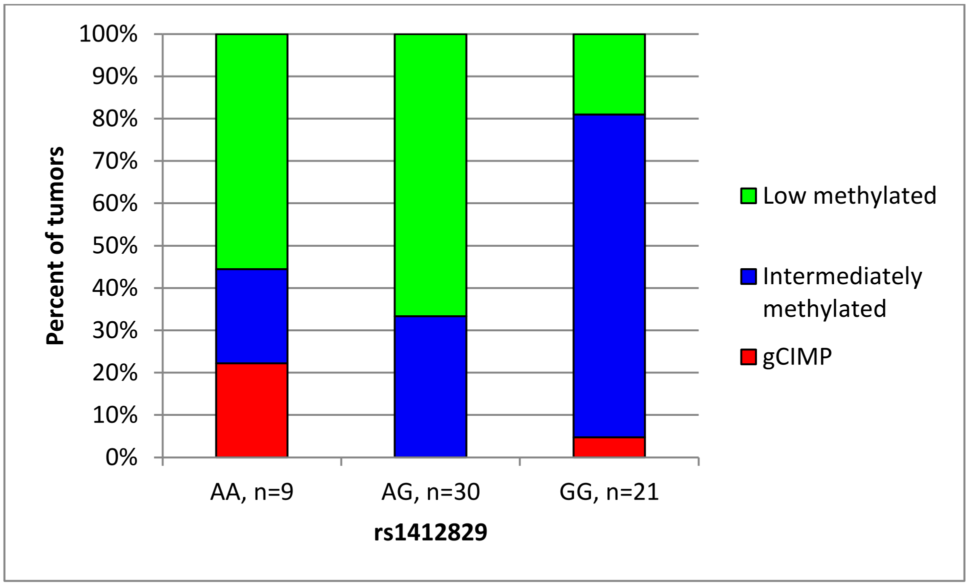

A

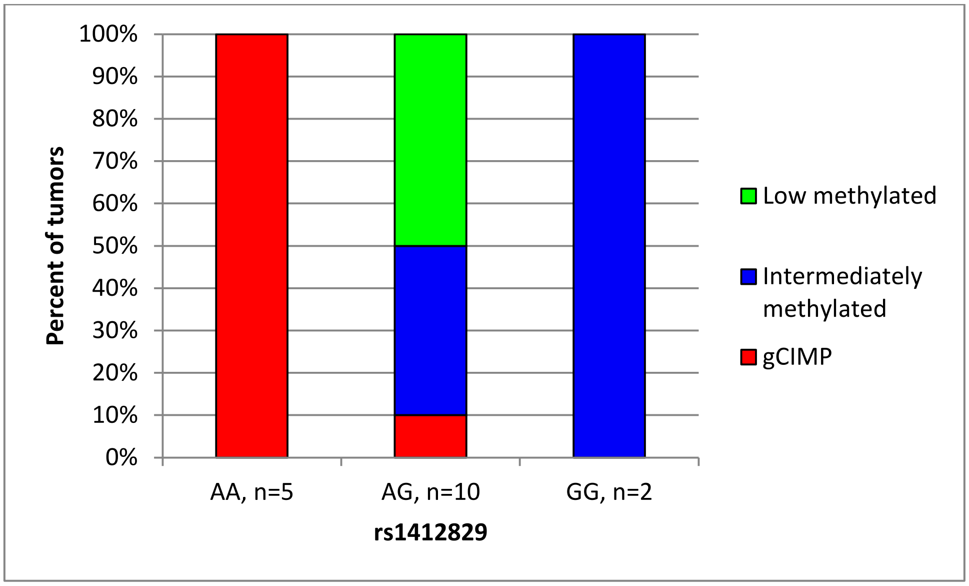

B

S3 Figure. Association between rs1412829 and DNA methylation pattern in glioblastoma and non-glioblastoma. (A) Glioblastoma (n=60) and (B) non-glioblastoma (lower grade astrocytoma and oligodendroglioma; n=17) tumors in the pilot dataset.
