# Supplementary material for: Relation between Established Glioma Risk Variants and DNA Methylation in the Tumor
Source: PLoS One. 2016 Oct 25;11(10):e0163067. doi: 10.1371/journal.pone.0163067 (PMC5079592; doi:10.1371/journal.pone.0163067)
Supplement: S1 Table — (DOCX) [file pone.0163067.s004.docx]

### **S1 Table.** All investigated SNPs and genes

| **snp** | **snp position (GRCh37/hg19)** | **risk allele** | **gene (transcript)** | **methylation sites within promoter, n** |
| --- | --- | --- | --- | --- |
| rs2736100 | chr5:1286516 | C | *TERT* (uc003jbz.1) | 4 |
|  |  |  | *TERT* (uc003jca.1) | 6 |
| rs2252586 | chr7:54978924 | T | *EGFR* (uc003tqh.3) | 2 |
|  |  |  | *EGFR* (uc011kco.2) | 2 |
|  |  |  | *EGFR* (uc011kcp.1) | 1 |
|  |  |  | *EGFR* (uc022ado.1) | 1 |
|  |  |  | *EGFR-AS1* (uc003tqo.4) | 1 |
|  |  |  | *SEC61G* (uc003tqf.3) | 9 |
| rs11979158 | chr7:55159349 | A | *EGFR* (uc003tqh.3) | 2 |
|  |  |  | *EGFR* (uc011kco.2) | 2 |
|  |  |  | *EGFR* (uc011kcp.1) | 1 |
|  |  |  | *EGFR* (uc022ado.1) | 1 |
| rs4295627 | chr8:130685457 | G | *CCDC26* (uc003ysq.2) | 3 |
|  |  |  | *FAM49B* (uc003ysw.4) | 3 |
|  |  |  | *FAM49B* (uc003yss.4) | 6 |
|  |  |  | *GSDMC* (uc003ysr.3) | 3 |
|  |  |  | *MYC* (uc003ysh.1) | 20 |
|  |  |  | *MYC* (uc003ysi.3) | 20 |
|  |  |  | *MYC* (uc003ysj.3) | 11 |
| rs1412829 | chr9:22043926 | G | *CDKN2A* (uc003zpk.3) | 1 |
|  |  |  | *CDKN2A* (uc003zpl.3) | 5 |
|  |  |  | *CDKN2B* (uc003zpn.3) | 3 |
|  |  |  | *CDKN2B*-AS1 (uc003zpm.3) | 2 |
|  |  |  | *MTAP* (uc003zph.3) | 2 |
| rs4977756 | chr9:22068652 | G | *CDKN2A* (uc003zpk.3) | 1 |
|  |  |  | *CDKN2A* (uc003zpl.3) | 5 |
|  |  |  | *CDKN2B* (uc003zpn.3) | 3 |
|  |  |  | *CDKN2B-AS1* (uc003zpm.3) | 2 |
| rs498872 | chr11:118477367 | A | *ARCN1* (uc001ptq.3) | 9 |
|  |  |  | *PHLDB1* (uc001ptr.2) | 7 |
|  |  |  | *PHLDB1* (uc001pts.3) | 8 |
|  |  |  | *PHLDB1* (uc001ptx.2) | 1 |
| rs6010620 | chr20:62309839 | G | *ARFRP1* (uc002ygc.4) | 11 |
|  |  |  | *RTEL1* (uc011abc.2) | 8 |
|  |  |  | *RTEL1* (uc002yfv.2) | 10 |
|  |  |  | *RTEL1-TNFRSF6B* (uc002yfx.1) | 5 |
|  |  |  | *STMN3* (uc002yfr.2) | 7 |
|  |  |  | *STMN3* (uc031ruo.1) | 8 |
|  |  |  | *TNFRSF6B* (uc002yfz.3) | 1 |
|  |  |  | *ZGPAT* (uc002ygi.2) | 12 |
|  |  |  | *ZGPAT* (uc002ygj.2) | 14 |
|  |  |  | *ZGPAT* (uc002ygn.4) | 13 |
| rs4809324 | chr20:62318220 | G | *ARFRP1* (uc002ygc.4) | 11 |
|  |  |  | *RTEL1* (uc011abc.2) | 8 |
|  |  |  | *RTEL1* (uc002yfv.2) | 10 |
|  |  |  | *RTEL1-TNFRSF6B* (uc002yfx.1) | 5 |
|  |  |  | *TNFRSF6B* (uc002yfz.3) | 1 |
|  |  |  | *ZGPAT* (uc002ygi.2) | 12 |
|  |  |  | *ZGPAT* (uc002ygj.2) | 14 |
|  |  |  | *ZGPAT* (uc002ygn.4) | 13 |
