# Supplementary material for: Relation between Established Glioma Risk Variants and DNA Methylation in the Tumor
Source: PLoS One. 2016 Oct 25;11(10):e0163067. doi: 10.1371/journal.pone.0163067 (PMC5079592; doi:10.1371/journal.pone.0163067)
Supplement: S2 Table — (DOCX) [file pone.0163067.s005.docx]

### S2 Table. Genes with no CpG probes in the promoter region

| **gene (transcript)** | **position^a^ (strand)** |
| --- | --- |
| *CDKN2B-AS1* (uc010miz.3) | chr9:22113664-22121093 (+) |
| *EGFR* (uc003tql.1) | chr7:55220082-55220357 (+) |
| *EGFR* (uc003tqn.3) | chr7:55232972-55233846 (+) |
| *GU228584* (uc022adp.1) | chr7:55277117-55279321 (+) |
| *MIR4457* (uc021xwd.1) | chr5:1309424-1309492 (-) |
| *Mir_384* (uc022bep.1) | chr9:22054370-22054454 (+) |
| *MTAP* (uc011lnl.2) | chr9:21818023-21865969 (+) |
| *SNORA25* (uc022bbm.1) | chr8:130880835-130880962 (-) |
| *PHLDB1* (uc001ptv.2) | chr11:118498020-118528748 (+) |
| *PHLDB1* (uc001ptw.2) | chr11:118500694-118528748 (+) |
| *PHLDB1* (uc010ryi.1) | chr11:118512268-118516501 (+) |
| *RTEL1-TNFRSF6B* (uc002yfy.3) | chr20:62326093-62330051 (+) |

^a^ GRCh37/hg19
