# Supplementary material for: Relation between Established Glioma Risk Variants and DNA Methylation in the Tumor
Source: PLoS One. 2016 Oct 25;11(10):e0163067. doi: 10.1371/journal.pone.0163067 (PMC5079592; doi:10.1371/journal.pone.0163067)
Supplement: S3 Table — (DOCX) [file pone.0163067.s006.docx]

### **S3 Table.** Associations between glioma risk SNPs and global DNA methylation.

|  |  |  | **pilot data set** | | |  | **TCGA^b^** | | |  |
| --- | --- | --- | --- | --- | --- | --- | --- | --- | --- | --- |
| **snp (risk allele)** | **annotation** | **geno-type** | **low methylated, n (%)** | **intermediately methylated, n (%)** | **high methylated (gCIMP), n (%)** | **p^a^** | **low methylated, n (%)** | **intermediately methylated, n (%)** | **high methylated (gCIMP), n (%)** | **p^a^** |
| rs2736100 (C) | *5p15.33 (TERT)* |  |  |  |  | 0.464 |  |  |  |  |
|  |  | AA | 9 (50.0) | 7 (38.9) | 2 (11.1) |  |  |  |  |  |
|  |  | AC | 19 (47.5) | 15 (37.5) | 6 (15.0) |  |  |  |  |  |
|  |  | CC | 6 (31.6) | 12 (63.2) | 1 (5.3) |  |  |  |  |  |
| rs2252586 (T) | *7p11.2* |  |  |  |  | 0.523 |  |  |  |  |
|  |  | CC | 20 (46.5) | 20 (46.5) | 3 (7.0) |  |  |  |  |  |
|  |  | CT | 9 (37.5) | 10 (41.7) | 5 (20.8) |  |  |  |  |  |
|  |  | TT | 2 (50.0) | 2 (50.0) | 0 (0.0) |  |  |  |  |  |
| rs11979158 (A) | *7p11.2 (EGFR)* |  |  |  |  | 0.835 |  |  |  |  |
|  |  | GG | 1 (50.0) | 1 (50.0) | 0 (0.0) |  |  |  |  |  |
|  |  | GA | 13 (52.0) | 9 (36.0) | 3 (12.0) |  |  |  |  |  |
|  |  | AA | 20 (40.0) | 24 (48.0) | 6 (12.0) |  |  |  |  |  |
| rs4295627 (G) | *8q24.21 (CCDC26)* |  |  |  |  | 0.945 |  |  |  |  |
|  |  | TT | 21 (46.7) | 19 (42.2) | 5 (11.1) |  |  |  |  |  |
|  |  | TG | 12 (41.4) | 13 (44.8) | 4 (13.8) |  |  |  |  |  |
|  |  | GG | 1 (33.3) | 2 (66.7) | 0 (0.0) |  |  |  |  |  |
| rs1412829 (G) | *9p21.3* (*CDKN2B*-AS1) |  |  |  |  | 8.07x10^-7^ |  |  |  | 0.137 |
|  |  | AA | 5 (35.7) | 2 (14.3) | 7 (50.0) |  | 69 (51.1) | 49 (36.3) | 17 (12.6) |  |
|  |  | AG | 25 (62.5) | 14 (35.0) | 1 (2.5) |  | 113 (59.8) | 66 (34.9) | 10 (5.3) |  |
|  |  | GG | 4 (17.4) | 18 (78.3) | 1 (4.3) |  | 40 (53.3) | 30 (40.0) | 5 (6.7) |  |
| rs4977756 (G) | *9p21.3* (*CDKN2B*-AS1) |  |  |  |  | 4.81x10^-5^ |  |  |  | 0.304 |
|  |  | AA | 8 (42.1) | 4 (21.1) | 7 (36.8) |  | 64 (50.0) | 50 (39.1) | 14 (10.9) |  |
|  |  | AG | 21 (61.8) | 12 (35.3) | 1 (2.9) |  | 116 (60.1) | 65 (33.7) | 12 (6.2) |  |
|  |  | GG | 5 (20.8) | 18 (75.0) | 1 (4.2) |  | 36 (50.7) | 29 (40.8) | 6 (8.5) |  |
| rs498872 (A) | *11q23.3* (*PHLDB1*) |  |  |  |  | 0.839 |  |  |  |  |
|  |  | GG | 13 (40.6) | 14 (43.8) | 5 (15.6) |  |  |  |  |  |
|  |  | GA | 17 (50.0) | 14 (41.2) | 3 (8.8) |  |  |  |  |  |
|  |  | AA | 4 (36.4) | 6 (54.5) | 1 (9.1) |  |  |  |  |  |
| rs6010620 (G) | *20q13.33* (*RTEL1*) |  |  |  |  | 0.700 |  |  |  |  |
|  |  | AA | 3 (60.0) | 2 (40.0) | 0 (0.0) |  |  |  |  |  |
|  |  | AG | 8 (33.3) | 12 (50.0) | 4 (16.7) |  |  |  |  |  |
|  |  | GG | 23 (47.9) | 20 (41.7) | 5 (10.4) |  |  |  |  |  |
| rs4809324 (G) | *20q13.33* (*RTEL1*) |  |  |  |  | 0.789 |  |  |  |  |
|  |  | AA | 26 (46.4) | 24 (42.9) | 6 (10.7) |  |  |  |  |  |
|  |  | AG | 8 (40.0) | 9 (45.0) | 3 (15.0) |  |  |  |  |  |
|  |  | GG | 0 (0.0) | 1 (100.0) | 0 (0.0) |  |  |  |  |  |

^a^ Chi-Square test or Fisher’s exact test (when expected sample count in a table cell was <5)

^b^ Only findings with p<0.05 in the pilot data set were investigated in the TCGA data set.

gCIMP, glioma CpG island methylator phenotype.
