# Supplementary material for: Relation between Established Glioma Risk Variants and DNA Methylation in the Tumor
Source: PLoS One. 2016 Oct 25;11(10):e0163067. doi: 10.1371/journal.pone.0163067 (PMC5079592; doi:10.1371/journal.pone.0163067)
Supplement: S4 Table — (DOCX) [file pone.0163067.s007.docx]

### **S4 Table.** Associations between glioma risk SNPs and CpG site methylation in nearby gene promoters

| **snp** | **risk allele** | **CpG probe** | **β-value^a^, mean** | | | **p^b^** |
| --- | --- | --- | --- | --- | --- | --- |
|  |  |  | **no risk allele** | **1 risk allele** | **2 risk alleles** |  |
| rs11979158 | A | cg01461514 | 0.49 | 0.36 | 0.37 | 0.755 |
| rs11979158 | A | cg02003682 | 0.80 | 0.81 | 0.79 | 0.688 |
| rs11979158 | A | cg16751451 | 0.32 | 0.30 | 0.29 | 0.773 |
| rs11979158 | A | cg18809076 | 0.61 | 0.47 | 0.44 | 0.504 |
| rs11979158 | A | cg20062492 | 0.69 | 0.72 | 0.74 | 0.907 |
| rs11979158 | A | cg22396409 | 0.13 | 0.12 | 0.12 | 0.975 |
| rs1412829 | G | cg00230302 | 0.07 | 0.08 | 0.08 | 0.947 |
| rs1412829 | G | cg00718440 | 0.17 | 0.09 | 0.09 | 0.144 |
| rs1412829 | G | cg02400248 | 0.12 | 0.07 | 0.07 | 0.680 |
| rs1412829 | G | cg03079681 | 0.08 | 0.06 | 0.05 | 0.883 |
| rs1412829 | G | cg07562918 | 0.21 | 0.20 | 0.21 | 0.136 |
| rs1412829 | G | cg10848754 | 0.39 | 0.09 | 0.11 | 0.732 |
| rs1412829 | G | cg13601799 | 0.15 | 0.09 | 0.09 | 0.374 |
| rs1412829 | G | cg13926295 | 0.19 | 0.11 | 0.10 | 0.454 |
| rs1412829 | G | cg14430974 | 0.44 | 0.07 | 0.10 | 0.290 |
| rs1412829 | G | cg19133618 | 0.12 | 0.08 | 0.08 | 0.772 |
| rs1412829 | G | cg25162921 | 0.36 | 0.29 | 0.29 | 0.657 |
| rs2252586 | T | cg00945507 | 0.29 | 0.31 | 0.20 | 0.404 |
| rs2252586 | T | cg01461514 | 0.34 | 0.40 | 0.44 | 0.442 |
| rs2252586 | T | cg02003682 | 0.80 | 0.80 | 0.81 | 0.971 |
| rs2252586 | T | cg03207732 | 0.05 | 0.05 | 0.05 | 0.484 |
| rs2252586 | T | cg06434455 | 0.02 | 0.02 | 0.02 | 0.307 |
| rs2252586 | T | cg08310974 | 0.07 | 0.07 | 0.08 | 0.526 |
| rs2252586 | T | cg09163442 | 0.07 | 0.07 | 0.08 | 0.880 |
| rs2252586 | T | cg10530063 | 0.05 | 0.08 | 0.02 | 0.343 |
| rs2252586 | T | cg13921000 | 0.03 | 0.02 | 0.02 | 0.092 |
| rs2252586 | T | cg15692229 | 0.84 | 0.83 | 0.89 | 0.478 |
| rs2252586 | T | cg16677535 | 0.09 | 0.08 | 0.11 | 0.078 |
| rs2252586 | T | cg16751451 | 0.29 | 0.31 | 0.30 | 0.527 |
| rs2252586 | T | cg17854265 | 0.09 | 0.09 | 0.10 | 0.491 |
| rs2252586 | T | cg18809076 | 0.45 | 0.47 | 0.53 | 0.823 |
| rs2252586 | T | cg20062492 | 0.74 | 0.73 | 0.69 | 0.500 |
| rs2252586 | T | cg22396409 | 0.12 | 0.12 | 0.12 | 0.992 |
| rs2736100 | C | cg02545192 | 0.32 | 0.29 | 0.33 | 0.756 |
| rs2736100 | C | cg03935379 | 0.59 | 0.62 | 0.56 | 0.311 |
| rs2736100 | C | cg07380026 | 0.68 | 0.67 | 0.63 | 0.355 |
| rs2736100 | C | cg10896616 | 0.12 | 0.15 | 0.15 | 0.874 |
| rs2736100 | C | cg11625005 | 0.43 | 0.48 | 0.53 | 0.259 |
| rs2736100 | C | cg17166338 | 0.81 | 0.79 | 0.79 | 0.890 |
| rs2736100 | C | cg19867481 | 0.74 | 0.75 | 0.73 | 0.705 |
| rs2736100 | C | cg23827991 | 0.77 | 0.73 | 0.68 | 0.001 |
| rs2736100 | C | cg25641025 | 0.66 | 0.68 | 0.69 | 0.756 |
| rs2736100 | C | cg26006951 | 0.49 | 0.49 | 0.50 | 0.811 |
| rs4295627 | G | cg00396059 | 0.01 | 0.01 | 0.00 | 0.125 |
| rs4295627 | G | cg01052743 | 0.07 | 0.07 | 0.05 | 0.303 |
| rs4295627 | G | cg01203549 | 0.04 | 0.04 | 0.04 | 0.196 |
| rs4295627 | G | cg01291833 | 0.11 | 0.11 | 0.11 | 0.523 |
| rs4295627 | G | cg02145916 | 0.52 | 0.49 | 0.51 | 0.915 |
| rs4295627 | G | cg02483101 | 0.41 | 0.43 | 0.36 | 0.805 |
| rs4295627 | G | cg03003858 | 0.01 | 0.00 | 0.00 | 0.007 |
| rs4295627 | G | cg03274021 | 0.09 | 0.11 | 0.09 | 0.606 |
| rs4295627 | G | cg03394237 | 0.07 | 0.06 | 0.06 | 0.375 |
| rs4295627 | G | cg03457528 | 0.07 | 0.11 | 0.06 | 0.477 |
| rs4295627 | G | cg04336279 | 0.91 | 0.94 | 0.89 | 0.194 |
| rs4295627 | G | cg04366381 | 0.01 | 0.01 | 0.00 | 0.636 |
| rs4295627 | G | cg04393391 | 0.04 | 0.04 | 0.03 | 0.701 |
| rs4295627 | G | cg05316065 | 0.50 | 0.49 | 0.45 | 0.862 |
| rs4295627 | G | cg05927173 | 0.07 | 0.06 | 0.06 | 0.635 |
| rs4295627 | G | cg06307601 | 0.09 | 0.12 | 0.09 | 0.685 |
| rs4295627 | G | cg08334153 | 0.08 | 0.12 | 0.08 | 0.856 |
| rs4295627 | G | cg08476224 | 0.23 | 0.20 | 0.10 | 0.573 |
| rs4295627 | G | cg09055355 | 0.05 | 0.05 | 0.05 | 0.973 |
| rs4295627 | G | cg10340053 | 0.05 | 0.05 | 0.05 | 0.970 |
| rs4295627 | G | cg10832291 | 0.01 | 0.01 | 0.00 | 0.274 |
| rs4295627 | G | cg10836034 | 0.07 | 0.07 | 0.06 | 0.406 |
| rs4295627 | G | cg11478273 | 0.15 | 0.17 | 0.14 | 0.399 |
| rs4295627 | G | cg12057615 | 0.05 | 0.04 | 0.04 | 0.443 |
| rs4295627 | G | cg12480416 | 0.11 | 0.12 | 0.08 | 0.805 |
| rs4295627 | G | cg12756521 | 0.02 | 0.04 | 0.00 | 0.631 |
| rs4295627 | G | cg13052755 | 0.94 | 0.94 | 0.94 | 0.983 |
| rs4295627 | G | cg13784855 | 0.09 | 0.12 | 0.06 | 0.424 |
| rs4295627 | G | cg14253951 | 0.09 | 0.09 | 0.09 | 0.695 |
| rs4295627 | G | cg15465092 | 0.00 | 0.00 | 0.00 | 0.475 |
| rs4295627 | G | cg17160660 | 0.08 | 0.08 | 0.04 | 0.518 |
| rs4295627 | G | cg17299732 | 0.11 | 0.10 | 0.13 | 0.639 |
| rs4295627 | G | cg18660971 | 0.04 | 0.06 | 0.00 | 0.144 |
| rs4295627 | G | cg19972619 | 0.06 | 0.06 | 0.06 | 0.330 |
| rs4295627 | G | cg20390814 | 0.94 | 0.94 | 0.95 | 0.391 |
| rs4295627 | G | cg22325480 | 0.14 | 0.22 | 0.07 | 0.067 |
| rs4295627 | G | cg23187103 | 0.00 | 0.00 | 0.00 | 0.197 |
| rs4295627 | G | cg23283234 | 0.07 | 0.09 | 0.07 | 0.919 |
| rs4295627 | G | cg23868848 | 0.09 | 0.11 | 0.09 | 0.950 |
| rs4295627 | G | cg24243265 | 0.90 | 0.90 | 0.94 | 0.230 |
| rs4295627 | G | cg24666276 | 0.10 | 0.13 | 0.10 | 0.019 |
| rs4295627 | G | cg25080152 | 0.09 | 0.09 | 0.11 | 0.106 |
| rs4295627 | G | cg26073844 | 0.79 | 0.75 | 0.78 | 0.544 |
| rs4295627 | G | cg26221243 | 0.07 | 0.07 | 0.07 | 0.729 |
| rs4295627 | G | cg26441142 | 0.07 | 0.07 | 0.07 | 0.617 |
| rs4295627 | G | cg26900458 | 0.09 | 0.14 | 0.10 | 0.191 |
| rs4295627 | G | cg27045396 | 0.02 | 0.02 | 0.01 | 0.781 |
| rs4295627 | G | cg27207274 | 0.08 | 0.08 | 0.07 | 0.260 |
| rs4809324 | G | cg00177013 | 0.07 | 0.07 | 0.06 | 0.399 |
| rs4809324 | G | cg01756902 | 0.11 | 0.18 | 0.09 | 0.238 |
| rs4809324 | G | cg02049682 | 0.76 | 0.80 | 0.69 | 0.284 |
| rs4809324 | G | cg03873930 | 0.72 | 0.76 | 0.83 | 0.484 |
| rs4809324 | G | cg04078896 | 0.21 | 0.21 | 0.43 | 0.266 |
| rs4809324 | G | cg04779788 | 0.00 | 0.00 | 0.00 | 0.653 |
| rs4809324 | G | cg05358404 | 0.68 | 0.63 | 0.87 | 0.042 |
| rs4809324 | G | cg09062397 | 0.10 | 0.10 | 0.08 | 0.223 |
| rs4809324 | G | cg09824849 | 0.38 | 0.37 | 0.30 | 0.377 |
| rs4809324 | G | cg12600109 | 0.88 | 0.90 | 0.75 | 0.279 |
| rs4809324 | G | cg13087314 | 0.87 | 0.87 | 0.86 | 0.630 |
| rs4809324 | G | cg13266717 | 0.01 | 0.01 | 0.01 | 0.991 |
| rs4809324 | G | cg14552772 | 0.01 | 0.01 | 0.00 | 0.198 |
| rs4809324 | G | cg16209860 | 0.56 | 0.55 | 0.38 | 0.394 |
| rs4809324 | G | cg16246590 | 0.01 | 0.02 | 0.02 | 0.038 |
| rs4809324 | G | cg16400469 | 0.00 | 0.00 | 0.00 | 0.176 |
| rs4809324 | G | cg16559570 | 0.67 | 0.70 | 0.60 | 0.426 |
| rs4809324 | G | cg17545146 | 0.00 | 0.00 | 0.01 | 0.549 |
| rs4809324 | G | cg17705079 | 0.02 | 0.01 | 0.03 | 0.341 |
| rs4809324 | G | cg18611245 | 0.25 | 0.23 | 0.19 | 0.921 |
| rs4809324 | G | cg19811473 | 0.08 | 0.05 | 0.04 | 0.448 |
| rs4809324 | G | cg20081540 | 0.84 | 0.84 | 0.88 | 0.488 |
| rs4809324 | G | cg20597143 | 0.55 | 0.59 | 0.53 | 0.567 |
| rs4809324 | G | cg20642413 | 0.29 | 0.32 | 0.42 | 0.352 |
| rs4809324 | G | cg21953717 | 0.94 | 0.96 | 0.96 | 0.170 |
| rs4809324 | G | cg22100873 | 0.19 | 0.13 | 0.22 | 0.078 |
| rs4809324 | G | cg22777832 | 0.08 | 0.08 | 0.09 | 0.859 |
| rs4809324 | G | cg23111772 | 0.02 | 0.02 | 0.00 | 0.269 |
| rs4809324 | G | cg24092470 | 0.08 | 0.05 | 0.09 | 0.072 |
| rs4809324 | G | cg24549085 | 0.04 | 0.04 | 0.03 | 0.447 |
| rs4809324 | G | cg24780981 | 0.01 | 0.01 | 0.01 | 0.518 |
| rs4809324 | G | cg25984973 | 0.10 | 0.10 | 0.09 | 0.794 |
| rs4809324 | G | cg26005165 | 0.00 | 0.00 | 0.01 | 0.781 |
| rs4809324 | G | cg27236539 | 0.01 | 0.01 | 0.01 | 0.470 |
| rs4977756 | G | cg00718440 | 0.18 | 0.09 | 0.09 | 0.181 |
| rs4977756 | G | cg02400248 | 0.12 | 0.07 | 0.07 | 0.424 |
| rs4977756 | G | cg03079681 | 0.08 | 0.06 | 0.05 | 0.996 |
| rs4977756 | G | cg07562918 | 0.21 | 0.20 | 0.21 | 0.215 |
| rs4977756 | G | cg10848754 | 0.39 | 0.08 | 0.11 | 0.715 |
| rs4977756 | G | cg13601799 | 0.16 | 0.09 | 0.09 | 0.065 |
| rs4977756 | G | cg13926295 | 0.19 | 0.11 | 0.10 | 0.391 |
| rs4977756 | G | cg14430974 | 0.45 | 0.07 | 0.11 | 0.136 |
| rs4977756 | G | cg19133618 | 0.13 | 0.08 | 0.08 | 0.661 |
| rs498872 | A | cg00145757 | 0.11 | 0.11 | 0.14 | 0.914 |
| rs498872 | A | cg00558376 | 0.06 | 0.05 | 0.05 | 0.745 |
| rs498872 | A | cg01864807 | 0.01 | 0.01 | 0.01 | 0.987 |
| rs498872 | A | cg04541078 | 0.06 | 0.05 | 0.05 | 0.019 |
| rs498872 | A | cg05207067 | 0.88 | 0.87 | 0.88 | 0.800 |
| rs498872 | A | cg05730269 | 0.54 | 0.59 | 0.58 | 0.487 |
| rs498872 | A | cg06290096 | 0.79 | 0.83 | 0.83 | 0.818 |
| rs498872 | A | cg08473858 | 0.28 | 0.28 | 0.26 | 0.733 |
| rs498872 | A | cg09426994 | 0.28 | 0.29 | 0.30 | 0.739 |
| rs498872 | A | cg11094953 | 0.00 | 0.01 | 0.01 | 0.501 |
| rs498872 | A | cg11286122 | 0.86 | 0.88 | 0.86 | 0.224 |
| rs498872 | A | cg14762010 | 0.03 | 0.03 | 0.02 | 0.165 |
| rs498872 | A | cg14826972 | 0.02 | 0.02 | 0.02 | 0.945 |
| rs498872 | A | cg16866038 | 0.01 | 0.01 | 0.01 | 0.566 |
| rs498872 | A | cg17470184 | 0.13 | 0.13 | 0.14 | 0.589 |
| rs498872 | A | cg17643109 | 0.03 | 0.03 | 0.02 | 0.241 |
| rs498872 | A | cg19006130 | 0.26 | 0.27 | 0.28 | 0.675 |
| rs498872 | A | cg20510012 | 0.74 | 0.81 | 0.83 | 0.275 |
| rs498872 | A | cg26475285 | 0.93 | 0.93 | 0.93 | 0.852 |
| rs498872 | A | cg26925644 | 0.54 | 0.59 | 0.53 | 0.445 |
| rs498872 | A | cg27488648 | 0.57 | 0.60 | 0.60 | 0.567 |
| rs6010620 | G | cg00177013 | 0.07 | 0.07 | 0.07 | 0.797 |
| rs6010620 | G | cg01756902 | 0.08 | 0.13 | 0.13 | 0.070 |
| rs6010620 | G | cg02049682 | 0.71 | 0.79 | 0.77 | 0.162 |
| rs6010620 | G | cg03524572 | 0.00 | 0.01 | 0.01 | 0.083 |
| rs6010620 | G | cg03873930 | 0.50 | 0.76 | 0.74 | 0.022 |
| rs6010620 | G | cg04013159 | 0.72 | 0.83 | 0.81 | 0.125 |
| rs6010620 | G | cg04078896 | 0.11 | 0.24 | 0.21 | 0.021 |
| rs6010620 | G | cg04757428 | 0.01 | 0.01 | 0.01 | 0.710 |
| rs6010620 | G | cg04779788 | 0.00 | 0.00 | 0.00 | 0.664 |
| rs6010620 | G | cg05358404 | 0.62 | 0.70 | 0.67 | 0.465 |
| rs6010620 | G | cg07382590 | 0.00 | 0.01 | 0.01 | 0.005 |
| rs6010620 | G | cg08291098 | 0.04 | 0.04 | 0.04 | 0.879 |
| rs6010620 | G | cg09062397 | 0.10 | 0.10 | 0.10 | 0.447 |
| rs6010620 | G | cg09824849 | 0.36 | 0.39 | 0.37 | 0.533 |
| rs6010620 | G | cg10397009 | 0.00 | 0.00 | 0.00 | 0.744 |
| rs6010620 | G | cg12600109 | 0.84 | 0.88 | 0.88 | 0.589 |
| rs6010620 | G | cg13087314 | 0.85 | 0.86 | 0.88 | 0.491 |
| rs6010620 | G | cg13266717 | 0.01 | 0.01 | 0.01 | 0.318 |
| rs6010620 | G | cg14552772 | 0.01 | 0.01 | 0.01 | 0.845 |
| rs6010620 | G | cg14862171 | 0.88 | 0.91 | 0.90 | 0.016 |
| rs6010620 | G | cg15559186 | 0.02 | 0.02 | 0.02 | 0.854 |
| rs6010620 | G | cg16209860 | 0.48 | 0.57 | 0.56 | 0.653 |
| rs6010620 | G | cg16246590 | 0.01 | 0.01 | 0.01 | 0.863 |
| rs6010620 | G | cg16400469 | 0.00 | 0.00 | 0.00 | 0.099 |
| rs6010620 | G | cg16559570 | 0.60 | 0.67 | 0.69 | 0.200 |
| rs6010620 | G | cg17296441 | 0.05 | 0.05 | 0.05 | 0.549 |
| rs6010620 | G | cg17545146 | 0.00 | 0.01 | 0.00 | 0.159 |
| rs6010620 | G | cg17705079 | 0.00 | 0.02 | 0.02 | 0.103 |
| rs6010620 | G | cg17953520 | 0.01 | 0.01 | 0.01 | 0.575 |
| rs6010620 | G | cg18611245 | 0.16 | 0.27 | 0.24 | 0.038 |
| rs6010620 | G | cg19811473 | 0.06 | 0.06 | 0.07 | 0.920 |
| rs6010620 | G | cg20081540 | 0.77 | 0.85 | 0.85 | 0.236 |
| rs6010620 | G | cg20597143 | 0.53 | 0.57 | 0.56 | 0.561 |
| rs6010620 | G | cg20642413 | 0.18 | 0.31 | 0.30 | 0.043 |
| rs6010620 | G | cg21953717 | 0.85 | 0.94 | 0.95 | 0.032 |
| rs6010620 | G | cg22100873 | 0.17 | 0.17 | 0.17 | 0.974 |
| rs6010620 | G | cg22777832 | 0.08 | 0.08 | 0.08 | 0.404 |
| rs6010620 | G | cg23111772 | 0.02 | 0.02 | 0.02 | 0.242 |
| rs6010620 | G | cg24092470 | 0.07 | 0.07 | 0.08 | 0.744 |
| rs6010620 | G | cg24549085 | 0.04 | 0.04 | 0.04 | 0.518 |
| rs6010620 | G | cg24780981 | 0.01 | 0.01 | 0.01 | 0.425 |
| rs6010620 | G | cg25984973 | 0.10 | 0.11 | 0.10 | 0.240 |
| rs6010620 | G | cg26005165 | 0.00 | 0.00 | 0.00 | 0.479 |
| rs6010620 | G | cg27236539 | 0.01 | 0.01 | 0.01 | 0.135 |

^a^ The β-value range from 0 to 1 for each cpg probe and tumor sample, where 0 indicates the absence DNA methylation and 1 indicates complete DNA methylation.

^b^ Kruskal-Wallis rank sum test
